# Social prediction errors in assisting strangers: the role of outcomes and contexts

**DOI:** 10.3389/fpsyg.2025.1516257

**Published:** 2025-08-19

**Authors:** Yan Li, Zhiwei Liu

**Affiliations:** School of Education and Psychological Science, Sichuan University of Science and Engineering, Zigong, Sichuan, China

**Keywords:** social prediction errors, helping behavior, social cognition, outcomes, contexts

## Abstract

**Introduction:**

Helping behavior is a fundamental aspect of social interaction, yet little is known about how accurately helpers can predict the emotional responses of help-seekers, particularly when interacting with strangers.

**Methods:**

This study investigated social prediction errors in helping behavior between strangers and examined how outcomes and contexts influence these errors. In three scenario-based experiments, we assessed helpers’ predictions and help-seekers’ evaluations of gratitude, satisfaction, enthusiasm, and competence across different helping situations.

**Results:**

Our findings reveal consistent underestimation of help-seekers’ positive emotions by helpers, with the degree of underestimation varying based on the outcomes and contexts of the helping behavior.

**Discussion:**

The results demonstrate that social prediction errors in helping behavior are context-dependent and outcome-based, highlighting the complexity of social cognition in prosocial behavior.

## 1 Introduction

Helping behavior is one of the most common and important characteristics of social relations. As an example of such behavior, attempts to help other people are made every day and in every place, even regarding people whom one has never met before (Malti and Dys, 2018). Instances of helping can vary from small tasks, such as giving directions or holding a door open for someone, to larger ones, such as comforting someone or intervening when someone needs help (Habashi et al., 2016). These normal and simple activities have been shown to enhance the wellbeing of both those who extend help and those who seek it. For instance, research has demonstrated that helping others in any way can have a positive effect on one’s mental health, enhance one’s satisfaction in life, and provide a sense of purpose (Curry et al., 2018; Hui et al., 2020). Moreover, helping even strangers is thought to aid in strengthening social bonds, social capital, and community cohesion (Twenge et al., 2007). Because helping behavior is commonly observed in various situations, it is essential to identify specific psychological factors that contribute to effective helping interactions and the barriers that may hinder successful helping outcomes.

One such barrier is a social prediction error, which we define in this context as a misjudgment or misestimation of another person’s emotional or behavioral response during or after a helping interaction (Zaki and Ochsner, 2012; Deri et al., 2019). These errors can occur on the part of either the help-seeker or the helper. For instance, help-seekers may inaccurately predict how likely others are to comply with their request or how much effort others are willing to give. Conversely, helpers may incorrectly anticipate the gratitude, satisfaction, or emotional response of the help-seeker.

A well-documented form of social prediction error is the underestimation-of-compliance effect, which refers to the consistent tendency of help-seekers to underestimate how willing others are to provide help (Bohns, 2016; Deri et al., 2019). The underestimation-of-compliance effect exists under various conditions, and the size of the effect is similar for both large requests (e.g., borrowing a cell phone) and small requests (e.g., asking for directions) (Flynn and Lake, 2008). It persists even when helping goes against social norms or expectations (Bohns et al., 2014) or when a request is repeated after an initial rejection (Newark et al., 2014). Additionally, the way a favor is requested can moderate this effect–direct requests are more effective than indirect ones (Cheshin et al., 2018), and the effect is more pronounced in individual cultures compared to collective cultures (Bohns et al., 2011). Help-seekers also tend to undervalue the effort others are willing to exert, partly due to a failure to appreciate the discomfort or guilt that helpers may feel when declining a request (Newark et al., 2017).

Several theories have been proposed to explain the underestimation-of-compliance effect. One explanation focuses on the role of interaction norms in helping behavior (Goldschmidt, 1998; Wee et al., 2022). Greenberg et al. (1971) suggest that both help-seekers and helpers are more likely to assess the costs and benefits associated with compliance. Social norms related to help-seeking and the motives behind helping behavior may lead help-seekers to exaggerate the magnitude of their request, while emotions such as humility and gratitude may cause them to believe that compliance has high costs (Flynn, 2003). Conversely, even helpers who make generous efforts to be polite must place an upper limit on the perceived effort or trouble taken to help, resulting in an undervaluation of the cost of compliance (Flynn, 2003). As a result, social interaction expectations may lead help-seekers and helpers to have opposing cognitive processes when evaluating the costs of compliance (Lens et al., 2018). Implicit social norms may cause help-seekers to perceive higher costs associated with compliance compared to helpers (McGuire, 2003), while helpers are more inclined to acknowledge the societal costs linked to declining the request (Lens et al., 2018).

The underestimation-of-compliance effect is primarily observed from the perspective of the help-seeker, although it reflects a broader category of social prediction errors. For clarity, we use the term social prediction error as an umbrella term to describe misalignments between one person’s prediction and another’s actual response in helping contexts. This includes the help-seeker’s underestimation of compliance as well as errors made by helpers in predicting the emotional responses of those they help. The present study focused on the errors made by helpers.

Although social prediction errors from help-seekers have been widely studied, researchers have only recently begun to examine whether helpers also make such errors when evaluating help-seekers’ emotional responses. A key study in this area is Shang et al. (2021), who investigated prediction errors in helping interactions between friends. In their study, participants read scenarios involving successful or unsuccessful help between friends and were asked to rate either their own or their friend’s emotional responses—such as gratitude, satisfaction, and the likelihood of seeking or offering help again. The findings showed that helpers systematically underestimated the gratitude, satisfaction, and positive evaluations they received from help-seekers, particularly when the help attempt was unsuccessful.

The present study builds directly on Shang et al.’s work by replicating their experimental design but extending it to the context of helping between strangers. While Shang et al. focused on helping behavior between friends—where intimacy may influence emotional judgments—our study shifts the focus to stranger interactions, where such intimacy is absent. Intimacy has been identified as a critical variable for social interaction and adaptation (Diener and Kansky, 2017), moderating attitudes toward individuals (Taillon et al., 2020). Moreover, individuals have a greater understanding of the minds and emotions of their friends compared to those of strangers (Ma-Kellams and Blascovich, 2012). Friends and strangers are also associated with different types of social capital, which may influence the dynamics of helping behavior. According to Perry et al. (2018), bonding capital refers to strong, close-knit relationships characterized by emotional support and trust, typically found among family and friends. In contrast, bridging capital involves weaker, more distant connections—such as those with strangers or acquaintances—that offer access to new information or opportunities. In helping interactions, friends tend to contribute to bonding capital by reinforcing emotional closeness, while strangers contribute to bridging capital by expanding one’s social network and potential resources (Perry et al., 2018). During social interactions with strangers, our behavior predictions can influence our own behavior, although these predictions are not always accurate (Fareri et al., 2020). Errors have been observed in predicting others’ thoughts and feelings (Karniol et al., 1997), and closeness can modulate the error effect (Deri et al., 2019). Thus, social prediction in the context of helping behavior may differ based on the level of intimacy between help-seekers and helpers, and findings from Shang et al. (2021) may not be generalizable to the context of helping behavior between strangers. However, it remains unknown whether helpers make social prediction errors when assisting strangers.

Social prediction errors pose a challenge when analyzing helping behavior between strangers, but addressing these errors is likely to yield higher-quality social interactions and relations. To communicate and work together effectively, individuals are expected to make accurate social judgments of others’ thoughts, feelings, and behaviors (Van Kleef et al., 2010). Such skills become critical for those who work with a variety of strangers, such as customer service, emergency relief, and community programs, as gaging the expectations and emotional responses of help-seekers can improve interactions (Fujiwara and Daibo, 2022). To reduce the possibility of embarrassment for helpers when implementing their helping strategies, there is a certain ‘ideal’ weight that helpers desire to meet in terms of approaches and actions of those seeking assistance (Dovidio et al., 2017). On the other hand, reducing social prediction errors can also enable help-seekers to explain their requirements more clearly and set reasonable expectations for possible assistance, leading to a more fruitful and useful encounter (Flynn and Bohns, 2015). In the end, addressing social prediction errors in helping contexts can contribute to creating a more responsive, compassionate, and harmonious society where people feel more at ease and confident asking for or providing aid to strangers in various circumstances.

The primary objective of this study was to address the issues present in the available literature and effectively tackle the problem of predicting behavior in helping situations among strangers. While previous research has investigated social prediction errors in the context of friends (Shang et al., 2021), the current study is among the first to focus on these phenomena within the context of helping strangers. Our purpose is to unveil different patterns and problems that are likely to arise when helping people who are unknown to the helper and with whom no prior relationship exists. We randomly assigned participants to either a help-seeker or a helper group and presented them with three different scenarios designed to illustrate contexts of strangers’ helping behavior. We also aimed to assess the accuracy of social prediction by introducing the consequences of helping, varying the success of the help, or request for help in the series of scenarios used. In three scenarios (train, photo, and bookstore), participants were asked to predict the subjective impressions of help-seeking individuals. Both sets of data include participants’ information about the mistakes (minor in the train scenario and major in the photo scenario) made by helpers. By including these typically neglected details in the design of this type of research, we aimed to cover other important facets that explore social prediction errors in helping situations.

Given the limited research on social predictions about strangers (Wakefield et al., 2019) and the challenges in estimating unfamiliar feelings or behaviors, we hypothesized that social prediction errors would occur during helping interactions between strangers. Specifically, we predicted that helpers would underestimate help-seekers’ gratefulness and satisfaction across scenarios and manipulated conditions. By investigating these hypotheses, our study aims to address a notable gap in the literature and provide new insights into the dynamics of social prediction in the context of helping behavior beyond close social relationships.

## 2 Methods

### 2.1 Participants

The sample size calculation was conducted using G*Power 3.1 software at 95% power, effect size of 0.25, and 0.05 significance level. This method suggested a minimal sample size of 210. A total of 240 volunteers (72 men, *M*_age_ = 20.54 years, *SD* = 1.72) were recruited for this study. All participants were undergraduate students from several large universities in China. All participants reported no mental health disorders in the last 6 months. Participants received a small amount of compensation for their participation.

### 2.2 Scenarios and design

Participants were provided with three hypothetical scenarios, which were classified based on the type of help provided: material help (train and photo scenarios) and psychological help (bookstore scenario). Detailed descriptions of each scenario, including the successful and unsuccessful conditions, are presented in Table 1. Participants were asked to imagine themselves as either help-seekers or helpers and to receive (help-seekers) or give (helpers) (un)successful assistance.

**Table 1:** Hypothetical scenarios used in this study.

Conditions	Scenarios
Material help: Train	
Successful	Imagine you (help-seeker version)/a stranger (helper version) were/was on a train, and you/the stranger needed to put your/her suitcase on the luggage rack. The suitcase was too heavy for you/the stranger to put it on the rack, so you/the stranger asked a stranger/you for help. The stranger/you would love to help and successfully put your/her suitcase on the luggage rack.
Unsuccessful	Imagine you (help-seeker version)/a stranger (helper version) were/was on a train, and you/the stranger needed to put your/her suitcase on the luggage rack. The suitcase was too heavy for you/the stranger to carry it on the rack, so you/the stranger asked a stranger/you for help. The stranger/You would love to help. However, the suitcase was also too heavy for the stranger/you. When he/you put the suitcase, he/you accidentally knocked the cup you/she placed on the table onto the ground, causing water to spill all over.
Material help: Photo	
Successful	Imagine you are traveling in a small town. You (help-seeker version)/A stranger (helper version) wanted to take photos for yourself/herself by using an iPhone. You/The stranger asked a stranger/you for help. The stranger/You would love to help and took several beautiful photos for you/the stranger.
Unsuccessful	Imagine you are traveling in a small town. You (help-seeker version)/A stranger (helper version) wanted to take photos for yourself/herself by using an iPhone. You/The stranger asked a stranger/you for help. The stranger/You would love to help. During the photography process, the stranger/you accidentally dropped the iPhone on the ground, causing it to break and be unable to power it on properly.
Psychological help: Bookstore	
Successful	Imagine you (help-seeker version)/a stranger (helper version) is walking on your/his way to a bookstore, but you/the stranger find(s) that you/he was lost. You/The stranger asked a stranger/you for help. The stranger/You would love to lead your/his way and bring you/him to the bookstore. You/The stranger finally get to the bookstore with his/your help.
Unsuccessful	Imagine you (help-seeker version)/a stranger (helper version) is walking on your/his way to a bookstore, but you/the stranger find(s) that you/he was lost. You/the stranger asked a stranger/you for help. The stranger/You would love to lead your/his way and bring you/him to the bookstore. However, the stranger/you led the way in the wrong direction, causing the bookstore to get further away.

The words in the parentheses are not shown in this study. Only a version of each scenario was given to each participant.

The three hypothetical scenarios are parallel and do not differ fundamentally. Moreover, similar to previous research (Shang et al., 2021), this study focuses on the prediction errors within each scenario, rather than the differences in prediction errors across different scenarios. Therefore, the three hypothetical scenarios do not serve as independent variables in this research. Thus, the study employed a 2 (Roles: Help-seeker, Helper) × 2 (Outcomes: Successful, Unsuccessful) between-subject design. Participants were randomly assigned to one of four groups.

### 2.3 Procedure

Participants were asked to read the three scenarios one by one. After reading each scenario, participants were instructed to make predictions (helpers) or evaluations (help-seekers) on several items. The items were borrowed from Shang et al. (2021). For example, helpers were asked to forecast how grateful the help-seekers would feel using the item: “Do you think the help-seeker is complaining or is thankful to you for your help?” (−7 = very complaining, 7 = very grateful). Correspondingly, help-seekers were asked about their feelings of gratitude using the item: “Are you complaining or grateful to the stranger helper?” (−7 = very complaining 7 = very grateful).

In total, participants responded to two items in the first two scenarios and four items in the last scenario, assessing various aspects of the helping interaction, such as gratitude, satisfaction, enthusiasm, and competence. All items were rated on a 7-point scale, with higher scores indicating more positive evaluations. The complete list of items used in the study is provided in the Supplementary Table 1.

## 3 Results

To determine the presence of social prediction errors in the context of stranger helping behavior, we employed two-way analyses of variance (ANOVA) to investigate the effects of roles (helpers vs. help-seekers) and outcomes (successful vs. unsuccessful) on various dependent variables for each scenario. In the simple effects analysis, we used *F*-tests to compare the means of helpers and help-seekers within each level of the outcome variable (successful vs. unsuccessful). The *F*-test is appropriate when comparing the means of two groups, as it tests the null hypothesis that the means of the two groups are equal. Although *t*-tests are commonly used for simple effects analysis, we chose to use *F*-tests because they are equivalent to *t*-tests when comparing two groups and provide a consistent presentation of results throughout the analysis. These analyses allowed us to examine whether there were significant differences between helpers’ predictions and help-seekers’ actual evaluations, indicating the presence of social prediction errors.

Descriptive statistics for predictions from helpers and evaluations from help-seekers under (un)successful conditions in scenarios are shown in Table 2.

**Table 2:** Means for predictions from helpers and evaluations from help-seekers under (un)successful conditions in scenarios.

Scenarios	Measures	Outcomes	Roles	
			Helper	Help-seeker
Material help: Train	Gratefulness	Successful	4.65 (2.73)	6.38 (1.52)
		Unsuccessful	0.52 (3.65)	2.45 (3.33)
	Satisfaction	Successful	4.72 (2.58)	6.35 (1.53)
		Unsuccessful	0.62 (3.57)	2.03 (3.12)
Material help: Photo	Gratefulness	Successful	4.83 (2.38)	6.33 (1.53)
		Unsuccessful	−1.43 (4.30)	−0.85 (3.27)
	Satisfaction	Successful	4.75 (2.34)	6.08 (1.68)
		Unsuccessful	−1.50 (4.17)	−1.13 (3.12)
Psychological help: Bookstore	Gratefulness	Successful	5.22 (2.49)	5.82 (1.90)
		Unsuccessful	−0.2 (4.06)	−1.15 (3.41)
	Satisfaction	Successful	5.07 (2.43)	6.37 (1.44)
		Unsuccessful	0.12 (3.69)	3.95 (3.21)
	Warmth	Successful	5.52 (2.01)	6.25 (1.42)
		Unsuccessful	3.93 (3.25)	−0.43 (3.23)
	Competence	Successful	4.72 (2.41)	6.38 (1.52)
		Unsuccessful	−1.08 (4.10)	0.03 (3.41)

The standard deviation of the mean is shown in parentheses.

### 3.1 Material help

#### 3.1.1 Train scenario

Significant main effects were found for both roles and outcomes on gratefulness and satisfaction. Help-seekers reported higher levels of gratefulness (*F* (1, 236) = 23.59, *p* < 0.001, $\eta_{p}^{2}$ = 0.09) and satisfaction (*F* (1, 236) = 17.74, *p* < 0.001, $\eta_{p}^{2}$ = 0.07) than helpers predicted. This indicates a general underestimation by helpers. Additionally, both helpers and help-seekers reported higher levels of gratefulness (*F* (1, 236) = 114.17, *p* < 0.001, $\eta_{p}^{2}$ = 0.33) and satisfaction (*F* (1, 236) = 135.12, *p* < 0.001, $\eta_{p}^{2}$ = 0.36) in successful helping situations compared to unsuccessful ones.

Significant interaction effects between the roles (helpers vs. help-seekers) and outcomes (successful vs. unsuccessful) for gratefulness (*F* (1, 236) = 21.45, *p* < 0.001, $\eta_{p}^{2}$ = 0.06) and satisfaction (*F* (1, 236) = 19.68, *p* < 0.001, $\eta_{p}^{2}$ = 0.04). Simple effects analyses showed that the underestimation effect was more pronounced in the successful condition for both gratefulness (*F* (1, 118) = 18.41, *p* < 0.001, $\eta_{p}^{2}$ = 0.13) and satisfaction (*F* (1, 118) = 17.76, *p* < 0.001, $\eta_{p}^{2}$ = 0.13), compared to the unsuccessful conditions—gratefulness (*F* (1, 118) = 9.19, *p* < 0.01, $\eta_{p}^{2}$ = 0.07) and satisfaction (*F* (1, 118) = 5.37, *p* < 0.05, $\eta_{p}^{2}$ = 0.04). Those results suggest that helpers tend to underestimate help-seekers’ gratitude and satisfaction more when their help is successful.

#### 3.1.2 Photo scenario

As in the Train scenario, significant main effects of roles and outcomes were found for both gratefulness and satisfaction. Help-seekers reported greater gratefulness (*F* (1, 236) = 7.00, *p* < 0.05, $\eta_{p}^{2}$ = 0.03) and satisfaction (*F* (1, 236) = 4.89, *p* < 0.05, $\eta_{p}^{2}$ = 0.02) compared to helpers’ predictions. Both groups also rated gratefulness (*F* (1, 236) = 291.88, *p* < 0.001, $\eta_{p}^{2}$ = 0.55) and satisfaction (*F* (1, 236) = 306.91, *p* < 0.001, $\eta_{p}^{2}$ = 0.57) higher in successful than in unsuccessful helping situations.

There were also significant interaction effects between roles and outcomes found for gratefulness (*F* (1, 236) = 18.47, *p* < 0.001, $\eta_{p}^{2}$ = 0.08) and satisfaction (*F* (1, 236) = 20.28, *p* < 0.001, $\eta_{p}^{2}$ = 0.12). Simple effects analyses revealed that helpers significantly underestimated both gratefulness (*F* (1, 118) = 16.89, *p* < 0.001, $\eta_{p}^{2}$ = 0.13) and satisfaction (*F* (1, 118) = 12.74, *p* < 0.001, $\eta_{p}^{2}$ = 0.10) in the successful condition. However, no significant underestimation effects were found in the unsuccessful condition for either gratefulness (*F* (1, 118) = 0.70, *p* > 0.05, $\eta_{p}^{2}$ = 0.01) or satisfaction (*F* (1, 118) = 0.3, *p* > 0.05, $\eta_{p}^{2}$ = 0.002). This suggests that helper’s misjudgments were more pronounced when their help was successful.

### 3.2 Psychological help

In the Bookstore scenario, the effects varied across the dependent variables. For gratefulness, only the outcome had a significant main effect (*F* (1, 236) = 242.73, *p* < 0.001, $\eta_{p}^{2}$ = 0.51); there was no main effect of roles (*F* (1, 236) = 0.19, *p* > 0.05, $\eta_{p}^{2}$ = 0.001), indicating no significant difference between helpers’ and help-seekers’ rating of gratefulness.

Regarding satisfaction (*F* (1, 236) = 49.44, *p* < 0.001, $\eta_{p}^{2}$ = 0.17), enthusiasm (*F* (1, 236) = 29.31, *p* < 0.001, $\eta_{p}^{2}$ = 0.11), and competence (*F* (1, 236) = 12.71, *p* < 0.001, $\eta_{p}^{2}$ = 0.05), significant main effects were found for roles. Moreover, the main effects of outcomes were also significant in satisfaction (*F* (1, 236) = 101.82, *p* < 0.001, $\eta_{p}^{2}$ = 0.30), enthusiasm (*F* (1, 236) = 151.7, *p* < 0.001, $\eta_{p}^{2}$ = 0.39), and competence (*F* (1, 236) = 242.29, *p* < 0.001, $\eta_{p}^{2}$ = 0.51). These findings indicate that help-seekers rated satisfaction, enthusiasm, and competence higher than helpers predicted, and that all participants rated these variables more positively in successful compared to unsuccessful helping situations.

Significant interaction effect between roles and outcomes were found for satisfaction (*F* (1, 236) = 12.04, *p* < 0.001, $\eta_{p}^{2}$ = 0.05) and enthusiasm (*F* (1, 236) = 57.74, *p* < 0.001, $\eta_{p}^{2}$ = 0.19), but not for gratefulness (*F* (1, 236) = 1.06, *p* > 0.05, $\eta_{p}^{2}$ = 0.002) and competence (*F* (1, 236) = 0.49, *p* > 0.05, $\eta_{p}^{2}$ = 0.002). Simple effects analyses showed that for satisfaction, helpers underestimated help-seekers’ feelings more in the unsuccessful condition (*F* (1, 118) = 36.71, *p* < 0.001, $\eta_{p}^{2}$ = 0.24) than in the successful condition (*F* (1, 118) = 12.74, *p* < 0.001, $\eta_{p}^{2}$ = 0.10). For enthusiasm, an interesting pattern emerged: helpers underestimated help-seekers’ evaluation in the successful condition (*F* (1, 118) = 5.34, *p* < 0.05, $\eta_{p}^{2}$ = 0.04), but overestimated it in the unsuccessful condition (*F* (1, 118) = 54.5, *p* < 0.001, $\eta_{p}^{2}$ = 0.32). Thus, helpers’ predictions were not only inaccurate but reversed in direction depending on the outcome.

## 4 Discussion

The present study investigated whether helpers make social prediction errors when assisting strangers, and how these errors vary depending on the type of help (Material vs. Psychological), outcomes, and context of helping behavior. Across three scenario-based experiments, we consistently found that helpers underestimated help-seekers’ emotional responses. However, the magnitude and direction of these errors depended on whether the help was successful or unsuccessful—highlighting the importance of interaction effects between roles and outcomes, as well as the type of help provided.

In the Material help scenarios (Train and Photo), helpers underestimated help-seekers’ gratitude and satisfaction, particularly when the help was successful. This pattern suggests that even when providing tangible assistance effectively, helpers may fail to appreciate the positive emotional impact of their actions on strangers. This finding contrasts with Shang et al. (2021), who reported stronger underestimation effects when helpers failed to assist their friends. The difference may reflect the role of relational closeness: people may be less attuned to strangers’ emotional response, especially when things go well, due to lower emotional investment or limited feedback (Deri et al., 2019). However, no underestimation effects were observed when an unsuccessful helping behavior caused big losses (e.g., the help-seeker’s iPhone was damaged in the Photo scenario). This suggests that significant negative outcomes may sharpen helpers’ awareness of consequences, reducing prediction errors. Helpers may attribute the failure to themselves and become more self-critical, leading to more accurate or even pessimistic estimates of how help-seekers feel (Buechel et al., 2017; Schneider et al., 2021).

In the Psychological help scenario (Bookstore), a different pattern emerged. No underestimation was found for gratitude, but a strong underestimation of satisfaction occurred in the unsuccessful condition. This discrepancy may be attributed to the nature of the helping task, which involved a longer and more complex effort compared to the Material help scenarios (Flynn and Lake, 2008). When help fails in such cases, helpers may overestimate their responsibility, while help-seekers may attribute the failure to external factors (e.g., the complexity of the route) (Zhao and Epley, 2021), leading to a gap in perception. Additionally, the distinct emotional nature of gratitude versus satisfaction may explain the discrepancy: gratitude tends to reflect perceived intent, while satisfaction is more outcome-dependent (Yu et al., 2018). Interestingly, helpers also underestimated enthusiasm in successful conditions but overestimated it when help was unsuccessful. This reversal may reflect self-serving biases or self-protective attributions—where unsuccessful helpers try to preserve their self-image by assuming their efforts were appreciated more than they actually were (Shepperd et al., 2008; Wang et al., 2017).

The findings emphasize that helpers’ predictions are context-dependent and influenced by the type of help provided. The presence of interaction effects suggests that the nature of the helping task and the emotional salience of the outcome shape how accurately helpers can predict others’ feelings. Helpers may have difficulty accurately understanding the emotional state of a stranger help-seeker due to an empathy gap (Zaki and Ochsner, 2012). Moreover, attribution biases (Bohns and Flynn, 2010) may contribute to helpers’ challenges in predicting help-seekers’ emotions accurately. The variability across scenarios also suggests that the type of helping behavior and the emotional salience of the outcome shape how accurately helpers can predict others’ feelings.

The findings of this study have several practical implications for understanding and improving social interactions in help contexts. First, the consistent underestimation of help-seekers’ positive feelings by helpers highlights the importance of promoting accurate social predictions. Misunderstandings about the impact of helping behavior on help-seekers’ emotions may lead helpers to undervalue their own efforts and hesitate to offer assistance in future (Flynn and Lake, 2008). To mitigate this, it may be beneficial to encourage perspective-taking and empathy among potential helpers, as well as to foster a culture of gratitude expression by help-seekers (Grant and Gino, 2010). Second, the findings highlight the importance of considering the type of help (Material vs. Psychological) and the potential costs and risks associated with helping behavior when examining social prediction errors. Helpers should be mindful of the impact of their actions on help-seekers’ emotions, particularly in situations involving psychological support (Zhang and Epley, 2009).

It is important to acknowledge the limitations of the current study, such as the hypothetical scenarios, student sample, and cultural specificity. The use of hypothetical scenarios may not fully capture the emotional richness and complexity of real-life helping situations. While using hypothetical scenarios is a common approach in social psychology research, it is essential to recognize that this method may lack the ecological validity of more naturalistic methods and may not fully reflect the complexity of real-life situations. Future research should explore social prediction errors in helping contexts using more naturalistic designs, such as experience diaries or field interventions. Future research also should explore social prediction errors in more diverse populations, using naturalistic designs and considering cultural factors. Additionally, investigating the mechanisms underlying these errors and developing interventions to mitigate their impact on helping behavior would be valuable. Moreover, future research should also investigate the role of personal variables, such as empathy, emotional intelligence, social support, and cultural background, in shaping social prediction errors in helping contexts. These individual differences may have a significant impact on how people interpret and predict others’ emotional reactions to helping behavior.

While the current study focuses primarily on the cognitive aspects of social prediction errors, such as predictions and attributions, it is essential to recognize the importance of the affective dimension in prosocial behavior. Emotions play a significant role in motivating and shaping helping behavior, and they may also influence the accuracy of social predictions in helping contexts (Bohns and Flynn, 2010). Future research should adopt a more balanced approach by investigating the interplay between cognitive and affective factors in social prediction errors. For instance, studies could examine how helpers’ and help-seekers’ emotional states, such as empathy, sympathy, or personal distress, influence their predictions and evaluations of helping outcomes (Eisenberg et al., 1991). Additionally, researchers could explore how the emotional valence of the helping situation (e.g., positive vs. negative) and the emotional intensity of the interaction affect the magnitude and direction of social prediction errors (County, 1987).

Previous research has highlighted the importance of emotional, cultural, and social factors in shaping prosocial behavior. For instance, studies have shown that empathy, social support, resilience, and emotional intelligence can significantly influence an individual’s willingness to help others and the effectiveness of their helping behavior (e.g., Mayer et al., 2008; Uchino, 2009). Incorporating these perspectives into the study of social prediction errors could help place our findings in a broader context of prosocial motivations and interpersonal understanding. Another important avenue for future research is to explore the relationship between prosociality and wellbeing, and how this relationship may influence social prediction errors in helping behavior. Studies using diary methods or repeated experiences have shown that engaging in prosocial behavior can lead to increased wellbeing, life satisfaction, and positive emotions (e.g., Aknin et al., 2018; Curry et al., 2018; Hui et al., 2020).

In conclusion, our study contributes to the understanding of social prediction errors in helping behavior between strangers, emphasizing the crucial role of outcomes and context. Helpers’ underestimation of help-seekers’ emotions is not uniform—it varies depending on whether help succeeds or fails. These findings highlight the need to promote accurate social judgments to support more satisfying and effective helping interactions.

## Data Availability

The datasets presented in this study can be found in online repositories. The names of the repository/repositories and accession number(s) can be found below: The data that supports the findings of this study is available in the Figshare repository: https://figshare.com/s/de0ecacbf36cb239f7f4.
