# Supplementary material for: Social prediction errors in assisting strangers: the role of outcomes and contexts
Source: Front Psychol. 2025 Aug 19;16:1516257. doi: 10.3389/fpsyg.2025.1516257 (PMC12402666; doi:10.3389/fpsyg.2025.1516257)
Supplement: Supplementary file 1 [file Supplementary_file_1.doc]

Supplementary Material: Items Used in the Study

Scenario 1: Train

Helpers:

1. Do you think the help-seeker is complaining or is grateful to you for your help? (-7 = very complaining, 7 = very grateful)
2. How satisfied do you think the help-seeker is with you for your help? (-7 = very dissatisfied, 7 = very satisfied)

Help-seekers:

1. Are you complaining or grateful to the stranger helper? (-7 = very complaining, 7 = very grateful)
2. How satisfied are you with the assistance from the stranger helper? (-7 = very dissatisfied, 7 = very satisfied)

Scenario 2: Photo

Helpers:

1. Do you think the help-seeker is complaining or is grateful to you for your help? (-7 = very complaining, 7 = very grateful)
2. How satisfied do you think the help-seeker is with you for your help? (-7 = very dissatisfied, 7 = very satisfied)

Help-seekers:

1. Are you complaining or grateful to the stranger helper? (-7 = very complaining, 7 = very grateful)
2. How satisfied are you with the assistance from the stranger helper? (-7 = very dissatisfied, 7 = very satisfied)

Scenario 3: Bookstore

Helpers:

1. Do you think the help-seeker is complaining or is grateful to you for your help? (-7 = very complaining, 7 = very grateful)
2. How satisfied do you think the help-seeker is with you for your help? (-7 = very dissatisfied, 7 = very satisfied)
3. How warmhearted do you think the stranger help-seeker will feel from your help? (1 = very cold, 7 = very warm)
4. How do you think the stranger help-seeker will feel about your competence for helping her/him? (1 = very poor, 7 = very good)

Help-seekers:

1. Are you complaining or grateful to the stranger helper? (-7 = very complaining, 7 = very grateful)
2. How satisfied are you with the assistance from the stranger helper? (-7 = very dissatisfied, 7 = very satisfied)
3. How warmhearted do you feel the stranger helper was? (1 = very cold, 7 = very warm)
4. How competent do you think the stranger helper was in providing assistance? (1 = very poor, 7 = very good)

Note: All items were rated on a 7-point scale, with higher scores indicating more positive evaluations.
